# Gene Expression System in Green Sulfur Bacteria by Conjugative Plasmid Transfer

**DOI:** 10.1371/journal.pone.0082345

**Published:** 2013-11-27

**Authors:** Chihiro Azai, Jiro Harada, Hirozo Oh-oka

**Affiliations:** 1 Department of Biological Sciences, Graduate School of Science, Osaka University, Toyonaka, Osaka, Japan; 2 Department of Medical Biochemistry, Kurume University School of Medicine, Kurume, Fukuoka, Japan; Belgian Nuclear Research Centre SCK/CEN, Belgium

## Abstract

Gene transfer and expression systems in green sulfur bacteria were established by bacterial conjugation with *Escherichia coli*. Conjugative plasmid transfer from *E. coli* S17-1 to a thermophilic green sulfur bacterium, *Chlorobaculum tepidum* (formerly *Chlorobium tepidum*) WT2321, was executed with RSF1010-derivative broad-host-range plasmids, named pDSK5191 and pDSK5192, that confer erythromycin and streptomycin/spectinomycin resistance, respectively. The transconjugants harboring these plasmids were reproducibly obtained at a frequency of approximately 10^-5^ by selection with erythromycin and a combination of streptomycin and spectinomycin, respectively. These plasmids were stably maintained in *C. tepidum* cells in the presence of these antibiotics. The plasmid transfer to another mesophilic green sulfur bacterium, *C. limnaeum* (formerly *Chlorobium phaeobacteroides*) RK-j-1, was also achieved with pDSK5192. The expression plasmid based on pDSK5191 was constructed by incorporating the upstream and downstream regions of the *pscAB* gene cluster on the *C. tepidum* genome, since these regions were considered to include a constitutive promoter and a ρ-independent terminator, respectively. Growth defections of the ∆*cycA* and ∆*soxB* mutants were completely rescued after introduction of pDSK5191-*cycA* and -*soxB* that were designed to express their complementary genes. On the other hand, pDSK5191-*6xhis-pscAB*, which incorporated the gene cluster of *6xhis-pscA* and *pscB*, produced approximately four times more of the photosynthetic reaction center complex with His-tagged PscA as compared with that expressed in the genome by the conventional natural transformation method. This expression system, based on conjugative plasmid, would be applicable to general molecular biological studies of green sulfur bacteria.

##  Introduction

Green sulfur bacteria (*Chlorobia*) are obligate anaerobic photoautotrophic bacteria [1,2]. They are commonly found in sulfur-rich anaerobic environments and grow by anoxygenic photosynthesis utilizing reduced sulfur compounds. The architecture of this photosynthetic system is much simpler than that of oxygenic photosynthesis of plant chloroplasts and cyanobacteria [3,4]; there is no thylakoid membrane-stacking structure in the cell, and the type-1 (Fe-S type) photosynthetic reaction center (RC) complex and menaquinol:cytochrome *c* oxidoreductase are supposed to form a linear photosynthetic electron transport chain within the inner cytoplasmic membrane [5,6]. Additionally, they have a lot of unique and uncommon features: for example, extraordinarily large antenna vesicles, chlorosomes [4], homodimeric structure of the RC complex [3], and inorganic carbon assimilation by the reductive TCA cycle [7,8]. Most of their features have been considered to be molecular-evolutionarily primitive [9,10]. Therefore, the photosynthesis of green sulfur bacteria is expected to be a model for the primeval light-energy conversion system and would provide important clues to understanding the evolutionary process to the present highly organized reaction apparatus in chloroplasts and cyanobacteria.

Green sulfur bacterial photosynthesis has been studied biochemically and spectroscopically since the 1950s, while its strictly anaerobic property has prevented the definitive understanding of its molecular architecture and reaction mechanism [3]. Advancement of the molecular genetic research on green sulfur bacteria has languished far behind other photosynthetic organisms until recently, and the chromosomal gene targeting method was accomplished in the thermophilic *Chlorobaculum tepidum* (formerly *Chlorobium tepidum*) in 2001 [11]. Whole-genome information also became available in 2002 [8]. Many of the gene-disrupted mutants isolated so far have revealed the detailed biosynthetic pathways of bacteriochlorophyll (BChl) *c* and of sulfur oxidation as well [4]. The gene expression system has also been constructed using *C. tepidum*, in which the *bchU* gene encoding the C-20 methyltransferase for BChl *c* biosynthesis was used as a platform for foreign genes. The *cruB* gene from *Chlorobium clathratiforme* strain DSM 5477 was inserted into the *bchU* locus and modified the carotenoid biosynthesis in *C. tepidum* [12]. However, the only acceptable photoautotrophic growth condition for *C. tepidum* has made it difficult to investigate any other photosynthetically essential and intriguing gene products, such as electron transfer components and carbon-assimilation enzymes. Gene transfer and expression methods, which enable the production of genetically modified proteins with epitope tags at the amino and/or carboxyl termini along with complementation of photoautotrophic growth, would be the most promising approach to finding a breakthrough in this situation. As a model case, we have recently developed a novel strategy to construct any site-directed mutants of the RC core protein by the insertional inactivation of the *recA* gene [13]. This strategy seems to be applicable in principle to any genes; however, manipulation based on the homologous DNA recombination requires much more time and effort to routinely obtain various mutants.

Another useful and convenient platform for gene expression experiments would thus be a plasmid vector that could shuttle between *Escherichia coli* and green sulfur bacteria. In 1995, T.M. Wahlund and M.T. Madigan demonstrated the conjugative transfer of broad-host-range plasmids from *E. coli* into *C. tepidum* and their maintenance in it [14]. Effective conjugations were confirmed especially by using derivative plasmids of the RSF1010 (IncQ group) [15]. However, other research groups that tried to introduce plasmids into *C. tepidum* according to their method could not obtain any stable transconjugants, unfortunately [16]. On the other hand, a large (~14 kbp) endogenous plasmid, pCL1, was isolated from the green sulfur bacterial species *C. thiosulfatiphilum* DSM 249 and was transferred to another species, *Chlorobium limicola* DSM 245 [17]. The recent extensive genome projects of 15 green sulfur bacterial strains have found that *Prosthecochloris aestuarii* DSM 271 harbors an endogenous plasmid, pPAES01 (~67 kbp) [18]. Although plasmids pCL1 and pPAES01 appeared to be potentially applicable as a simple transformation method in green sulfur bacteria, any shuttle vectors derived from them have not yet been developed.

In this paper, we report the gene expression system for *C. tepidum* using an RSF1010-derivative conjugative plasmid. The plasmid was reproducibly transferred under ordinary conjugation conditions and was maintained stably after continuous cultivation in the presence of antibiotics. Then, the expression plasmid was constructed based on this conjugative plasmid, which worked well for gene complementation experiments and protein productions in *C. tepidum*. The availability of this system was also confirmed in another species, *C. limnaeum* (formerly *Chlorobium phaeobacteroides*) RK-j-1. It would be a general molecular genetic tool in green sulfur bacteria and also become an effective way to express even fragile proteins that are stable only under anaerobic conditions.

## Materials and Methods

### Bacterial Strains and Culture Conditions

Bacterial strains used in this study are listed in Table 1. *C. tepidum* WT2321 [14] and *C. limnaeum* RK-j-1 [19] were used as the wild-type strains in the present study. Cultivation of *C. tepidum* and *C. limnaeum* in liquid CL media and on solid CP plates was routinely performed in essentially the same fashion as previously described [11]. Growth temperature was set at 40°C for *C. tepidum* and at 30°C for *C. limnaeum*, and the intensity of illumination was adjusted to 30 µmol photons/m^2^/s with incandescent lamps unless otherwise specified. The *E. coli* DH5α strain [20] was routinely used for molecular cloning to construct plasmids. The *E. coli* S17-1 strain [21], which has the IncPα-group plasmid RP4-2 integrated in the chromosome, was used as a donor for conjugative plasmid transfer experiments. *E. coli* cells were grown in the liquid or on solid LB media at 37°C.

**Table 1:** Bacterial strains used in this study.

**Bacterial strain**	**Genotype or description**	**Source or reference**
*E. coli*		
DH5α	F^-^ Φ80*lacZ*ΔM15 Δ(*lacZYA-argF*)U169 *deoR recA1 endA1 hsdR17*(r_K_^-^ m_K_^+^) *phoA supE44* λ^-^ *thi-1 gyrA96 relA1*	[20]
S17-1	*pro thi hsdR17*(r_K_^-^ m_K_^+^) *recA* RP4-2(Tc^r^::Mu-Km^r^::Tn7)	[21]
*C. tepidum*		
WT2321	Plating strain derivative of the wild-type strain TLS-1	[14]
∆*recA*	∆*recA::aacC1*(Gm**^*r*^**); a mutant strain incapable of homologous recombination	[13]
∆*cycA*	∆*cycA::aadA*(Sm^r^/Sp^r^); a mutant strain lacking periplasmic cytochrome *c*-554	[28]
∆*soxB*	∆*soxB::aacC1*(Gm**^*r*^**); a mutant strain incapable of thiosulfate oxidation	[5]
6HA	∆*pscA*::(*aadA-6xhis-pscA*); a mutant strain expressing 6xHis-tagged PscA	This study
6HA-∆*recA*	∆*recA::aacC1*(Gm**^*r*^**) ∆*pscA*::(*aadA-6xhis-pscA*); a double mutant strain of ∆*recA* and 6HA	This study
*C. limnaeum*		
RK-j-1	Plating strain derivative of the wild-type strain 1549	[19]

The following antibiotics with specified concentrations were added to the media if required: 30 µg/mL of gentamicin, 1 µg/mL of erythromycin, or a mixture of 100 µg/mL of streptomycin and 50 µg/mL of spectinomycin for *C. tepidum*; a mixture of 100 µg/mL of streptomycin and 150 µg/mL of spectinomycin for *C. limnaeum*; 100 µg/mL of ampicillin, 25 µg/mL of kanamycin, 30 µg/mL of streptomycin, 100 µg/mL of spectinomycin, or 200 µg/mL of erythromycin for *E. coli*. 

### Construction of the Conjugative Plasmid

**Figure 1:**
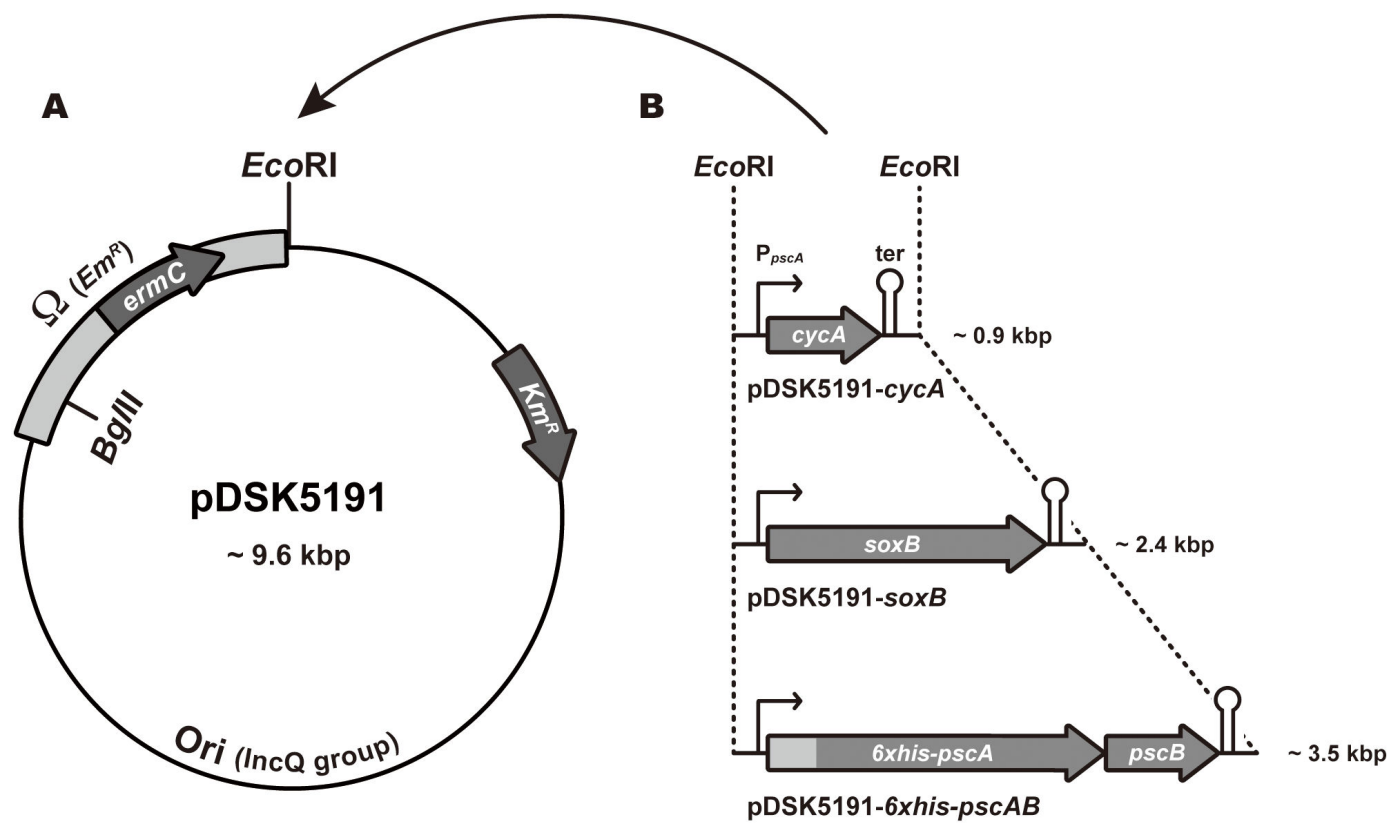
Schematic genetic maps of pDSK5191 and its derivative expression plasmids. (A) Genetic map of the IncQ-group conjugation plasmid pDSK5191. Protein coding sequences are shown as block arrows. The pale gray rectangle represents the region of Ω-cassette in which T4-phage transcription and translation terminator sequences [23] are located at both ends. “Ori” represents the region containing the *oriV* and *oriT* sequences, which are derived from RSF1010 and are required for replication and mobilization of the plasmid, respectively [15]. (B) Expression constructs of the pDSK5191-derivative plasmids. Each construct was inserted into the unique *Eco*RI site of pDSK5191, as indicated by the arch-shaped arrow at the top of the panels. Protein-coding sequences are shown as block arrows. The pale gray rectangle represents the six-consecutive histidine-tag (6xhis) attached to the 5’ end of the *pscA* gene. “P_*pscA*_” and “ter” are a putative constitutive promoter and a ρ-independent transcription terminator, respectively, of the *C. tepidum* *pscAB* gene cluster.

Major plasmids used in this study are listed in Table 2. All PCR primers used in the present study are listed in Table S1 along with their complete sequences. The conjugation plasmid was constructed based on the RSF1010-derivative conjugation plasmid pDSK519 [22], which was a generous gift from Dr. N.T. Keen (University of California, Riverside). The construction schemes were summarized in Figure S1. The blunted *Bam*HI digest of pDSK519 was ligated with the *ermC* gene- and Ω-teminator-containing *Pvu*II fragment of pUCEm, which was a generous gift from Dr. M. Ishiura (Nagoya University, Japan), in such a way as to arrange the *ermC* gene and the *lac* promoter on pDSK519 in the same direction, yielding pDSK5191 (Figure 1A). In the same manner, the *aadA* gene-containing *Sma*I fragment of pHP45Ω [23] was inserted into the blunted *Bam*HI site of pDSK519, yielding pDSK5192. 

**Table 2:** Plasmid used in this study.

**Plasmid**	**Relevant characteristics**	**phenotype**	**Source or reference**
pDSK519	A derivative conjugative plasmid of RSF1010 (IncQ)	Km^r^	[22]
pDSK5191	A derivative of pDSK519, containing an Em^r^ cassette of pUCEm	Km^r^ Em^r^	This study
pDSK5191A	A derivative of pDK5191, containing the *cat* and *ccdB* genes flanked by the *attR1* and *attR2* sequences	Km^r^ Em^r^ Cm^r^ *ccdB* **^*+*^**	This study
pDSK5191-*cycA*	A derivative of pDK5191, expressing the *cycA* gene with the P_*pscA*_ promoter	Km^r^ Em^r^	This study
pDSK5191-*soxB*	A derivative of pDK5191, expressing the *soxB* gene with the P_*pscA*_ promoter	Km^r^ Em^r^	This study
pDSK5191-*6xhis-pscAB*	A derivative of pDK5191, expressing the *6xhis-pscAB* gene with the P_*pscA*_ promoter	Km^r^ Em^r^	This study
pDSK5192	A derivative of pDSK519, containing a Sm^r^/Sp^r^ cassette of pHP45Ω	Km^r^ Sm^r^ Sp^r^	This study
PUCEm	Contains the *ermC* gene flanked by Ω-terminators	Ap^r^ Em^r^	M. Ishiura
pHP45Ω	Contains the *aadA* gene flanked by Ω-terminators	Ap^r^ Sm^r^ Sp^r^	[23]
pHP45-HisA	Contains the DNA construct to make the *C. tepidum* 6HA mutant strain	Ap^r^ Gm**^*r*^**	This study
pHP45-HisAB	Contains the *6xhis-pscAB* gene	Ap^r^	[13]
pENTR-PT	Contains the P_*pscA*_ promoter and terminator between the *attL1* and *attL2* sequences	Km^r^	This study
pENTR-cycA	Contains an expression construct for the *cycA* gene between the *attL1* and *attL2* sequences	Km^r^	This study
pENTR-soxB	Contains an expression construct of the *soxB* gene between the *attL1* and *attL2* sequences	Km^r^	This study
pENTR-HisAB	Contains an expression construct of the *6xhis-pscAB* gene between the *attL1* and *attL2* sequences	Km^r^	This study

To facilitate the construction of expression plasmids of *C. tepidum*, pDSK5191 was modified for conversion into a destination plasmid using Invitrogen’s Gateway® system [24]. In this system, a region between the *attL1* and *attL2* sequences on an entry plasmid is specifically exchanged with a region between the *attR1* and *attR2* sequences on a destination plasmid, which enables ligation-independent subcloning from an entry plasmid to a destination plasmid. Indeed, the RfA fragment of the Gateway® Vector Conversion Kit (Invitrogen) was inserted into the blunted *Eco*RI digest of pDSK5191 in such a way that the direction from *attR1* toward *attR2* was the same as those of the *ermC* gene and the *lac* promoter on pDSK5191, yielding the destination plasmid pDSK5191A. 

On the other hand, the entry plasmid containing the expression construct was constructed by exploiting the promoter and terminator of the *C. tepidum pscAB* gene cluster. A 3.4 kbp fragment amplified from genomic DNA with pscB-996F and pscA-4435R was cloned into the blunt-ended *Bam*HI site of pHP45, yielding plasmid pHP45-AB-PT. After *Hin*dIII digestion, a 1.1-kbp fragment of pHP45-AB-PT containing the terminator of the *pscAB* gene cluster was ligated with the 4.8-kbp *Hin*dIII fragment of pHP45-HisAB [13] in the proper direction, yielding plasmid pHP45-HisAB-PT. The *Eco*RI fragment containing the 6x*his-pscAB* gene cluster of pHP45-HisAB-PT was ligated with the *Eco*RI digest of pENTR1A (Invitrogen) in such a way that the direction from *attL1* toward *attL2* was the same as that of the *6xhis-pscAB* gene cluster, yielding pENTR-HisAB. Then, using pENTR-HisAB as a template, PCR was performed with the primers pscB-1100R (*Blp*I) and pscA-2448F followed by digestion with *Nco*I. The resultant fragment was ligated with the multiple cloning site-containing *Nco*I-*Stu*I fragment of pKF3 (TaKaRa, Japan), yielding a unique entry plasmid, pENTR-PT, generally useful for the present study. This plasmid has approximately 450-bp upstream and 100-bp downstream regions of the *pscAB* gene cluster as constitutive promoter- and ρ-independent terminator-including regions, respectively, and the partial multiple cloning sites derived from pKF3 between the promoter and terminator, in which the *Nco*I site carried a translational ATG initiation codon. In order to construct the entry plasmid containing the *C. tepidum cycA* and *soxB* genes, their coding regions were amplified from genomic DNA by PCR using their specific primer sets: cycA-2684F and cycA-3039R (*Blp*I) for the *cycA* gene, and soxB-4306F and soxB-6170R (*Blp*I) for the *soxB* gene. Each amplified fragment was digested with *Blp*I and ligated with the *Blp*I digest of the linearized pENTR-PT, which was pre-amplified by PCR using the primers pscB-1100R (*Blp*I) and pscA-4070F, yielding pENTR-cycA and pENTR-soxB. The expression constructs on the resultant entry plasmids were finally subcloned into pDSK5191A by the LR-recombination reactions in the Gateway® system, yielding the three kinds of expression plasmids in *C. tepidum*: pDSK5191-*cycA*, pDSK5191-*soxB*, and pDSK5191-*6xhis-pscAB* (Figure 1B).

### Conjugative Plasmid Transfer Experiments

Conjugation experiments between *E. coli* S17-1 and *C. tepidum* or *C. limnaeum* were performed by the previously described biparental method [14] with minor modifications. The overnight culture of the donor *E. coli* S17-1 cells was diluted 10^-1^ with an LB medium containing no antibiotics and incubated at 37°C for 2 hours with vigorous agitation. After being washed and resuspended in fresh LB media, it was used for the donor culture. *C. tepidum* and *C. limnaeum* recipient cultures were grown in a CL medium without antibiotics until the late-exponential phase, in which the optical density at 660 nm (O.D._660_) was attained at 1.5–2.0. 500 µL of the donor culture and an equal volume of the recipient culture were mixed in a 1.5-mL microcentrifuge tube and were spun down. After being resuspended in 200 µl of the CPC medium (a liquid CP medium supplemented with 0.05% yeast extract), the parental cell mixture was brought into the anaerobic glove box (Coy Laboratory, U.S.A.) and was spotted onto the CPC plate (a solid CP plate supplemented with 0.01% yeast extract) by 50-µL aliquots. For the mating, the CPC plate was placed in an anaerobic jar without a sulfide-generating system and incubated under illumination for 16–20 hours at 37°C for *C. tepidum* and for 3–5 days at 30°C for *C. limnaeum*. The four spots, in the present study, were scraped up and resuspended into 1.0 mL of a CL medium and spread onto the selective CP plate containing the appropriate antibiotics. The selection plate was placed in an anaerobic jar with a sulfide-generating system and incubated under illumination. Transconjugant colonies usually appeared within one or two weeks, and their liquid cultures were obtained by inoculating them into CL media. 

Conjugation frequencies were estimated as the colony-forming frequencies on the selective CP plates. The mixtures that were recovered after mating were spread onto both selective and non-selective CP plates, and the colonies that appeared on these plates were counted. The colony-forming frequencies were calculated as the ratios of the number of colonies on the selective plates to that on the non-selective plates. The average value and standard deviation for each mating condition were obtained from at least three independent experiments. Contamination of the parent *E. coli* S17-1 cells in the transconjugant cultures was examined by spreading the CL cultures onto LB plates. Plasmid DNA in the *C. tepidum* and *C. limnaeum* cells was isolated from the CL cultures by the conventional alkaline-SDS method [25] with one minor modification: After adding a potassium acetate solution to the cell lysate, a 10^-1^ volume of chloroform was added to the sample and mixed vigorously to remove excess amounts of pigments. Clear cell lysate was obtained in an aqueous phase after centrifugation.

### Construction of *C. tepidum* *strain* 6HA

The stable *C. tepidum* mutant strain 6HA, expressing His-tagged PscA polypeptides, was constructed by natural transformation. The plasmid was constructed as follows: A 1.0-kbp upstream region of the *C. tepidum pscAB* gene cluster was amplified from the genomic DNA by PCR using the primers pscA-4072F (*Nco*I) and dapF-5115R (*Sma*I) and ligated with the *Sma*I digest of pUC118 (TaKaRa, Japan). The resultant plasmid was linearized by PCR using the primers pscA-4094R and pscA-4095F and ligated with the *aadA* cassette amplified from pHP45Ω with the primers aadA-F2 (*Sma*I) and aadA-R2 (*Sma*I, *Blp*I, *Hin*dIII), yielding pUC118-P_*pscA*_::*aadA*. The P_*pscA*_::*aadA* region was amplified by PCR using the primers pscA-4072F (*Nco*I) and dapF-4676R, and the N-terminal side of the *6xhis-pscA* gene was amplified from pHis15b [13] by PCR using the primer pscA-3561F and the T7 Promoter primer. These two fragments were digested with *Nco*I and ligated simultaneously into the *Sma*I site of pHP45, yielding pHP45-HisA. After checking the sequence, the PCR-amplified fragment by the primers HP45-blaF and HP45-ropR was used as the DNA construct for the natural transformation.

Natural transformation of *C. tepidum* was performed according to the previously described method [11]. Streptomycin/spectinomycin-resistance clones were repeatedly streaked on selective CP plates several times until the mutant allele was fully segregated. Segregation of the allele was confirmed by PCR using the primers pscA-2448F and dapF-5291R, whose annealing sequences were located outside the homologous recombination regions. For the preliminary screening of the transformant colonies on the plates, the colony PCR method was carried out. The fully segregated mutant alleles of the transformants were then confirmed by direct DNA sequencing of the PCR products obtained after growing positive clones in liquid media. To construct the 6HA-∆*recA* strain, the *recA* gene was disrupted in the 6HA strain as described previously [13].

### Growth Measurements of *C. tepidum*

The growth of *C. tepidum* was measured in the CL medium by monitoring the optical density at 660 nm (O.D._660_), essentially as described previously [5]. The growth temperature was set at 40°C for the measurements in the presence of antibiotics. The doubling times were calculated by linear regression analyses for the semilog plots of the O.D._660_ values against the incubation times in the mid-to-late-exponential-growth phase (0.5 < O.D._660_ < 1.5) in order to avoid overestimation by the transient secretion of elemental sulfur globules in the early-to-mid-exponential phase [5,26]. The final cell yields were estimated by the O.D._660_ values obtained after 60-hour cultivation. The average values and standard deviations were obtained from at least three independent experiments.

### Isolation of the His-tagged RC Complex from *C. tepidum* Membranes

The solubilization of *C. tepidum* membranes was carried out on a small scale as follows: All procedures were performed at room temperature under aerobic and dim-light conditions unless otherwise specified. The cells (about 0.3 g wet weight), which were recovered from ordinary CL cultures grown in 30-mL test tubes, were suspended in 1 mL of buffer A (50 mM Tris-HCl (pH 8.0), 1 mM EDTA, 2 mM DTT, and protease inhibitors) and were packed into 2.0-mL microcentrifuge tubes containing zircon-silica beads (diameter = 0.1 mm, TOMY). The microcentrifuge tubes were then vigorously agitated at 4,500 rpm for 30 seconds at 4°C five times (BSP-3110BX, BioSpec) in order to disrupt the cells by causing them to collide with the beads. After the undisrupted cells were removed by centrifugation at 1,600×*g* for 15 min at 4°C, the supernatant lysates were ultracentrifuged at 100,000×*g* for 60 min at 4°C. The resultant pellets were resuspended in 500 µL of buffer A, which usually resulted in 600 µL suspensions of the chlorosome-containing crude membranes at ca. 5.0 mg of total BChls *a* and *c* per mL. The suspensions were mixed with equal volumes (600 µl) of buffer S (60 mM *n*-octyl-β-D-glucoside (Sigma-Aldrich), 600 mM NaCl, 50 mM Tris-HCl (pH 8.0), 1 mM EDTA, 2 mM DTT, and protease inhibitors) in 1.5-mL microcentrifuge tubes and were gently shaken for 60 min. After ultracentrifugation at 100,000×*g* for 60 min at 4°C, the supernatants were collected as extracts containing detergent-solubilized RC complexes.

His-tagged RC complex was recovered from the extracts by small-scale Ni^2+^-affinity purification. The extracts (~1.2 µL) were mixed with 100-µl aliquots of the pre-equilibrated Ni^2+^-immobilized resin His-Accept (Nacalai Tesque, Inc., Japan), followed by gentle shaking in 1.5-mL microcentrifuge tubes for 60 min. After washing the resin twice with 1 mL of buffer W (1 mM sucrose monolaurate, 5 mM Imidazole, 300 mM NaCl, 50 mM Tris-HCl (pH 8.0), and 2 mM DTT), the adsorbed His-tagged RC complex was eluted with 500 µl of buffer E (1 mM sucrose monolaurate, 300 mM Imidazole, 300 mM NaCl, 50 mM Tris-HCl (pH 8.0), and 2 mM DTT).

### Optical Estimations of the RC Contents

The RC contents were estimated by measuring chemically induced redox difference absorption changes at 840 nm, whose amplitudes reflected the contents of a special pair of BChls *a*, the primary electron donor P840. The differential extinction coefficient for P840 was assumed to be 100 mM^-1^cm^-1^ at 830 nm [27]. The samples were oxidized by the addition of small amounts of ferricyanide and were then reduced by the addition of excess amounts of ascorbate. The sample solutions were adequately diluted with buffer M (1 mM sucrose monolaurate (Dojindo, Japan), 50 mM Tris-HCl (pH 8.0), 1 mM EDTA, and 2 mM DTT) as necessary. The average values and standard deviations of the RC contents were obtained from at least three independent cultures. Absorption spectra were measured by a UV-visible spectrophotometer, UV-3101PC (Shimadzu, Japan).

## Results

### Conjugative Plasmid Transfer to *C. tepidum*

Plasmids pDSK519 and pGSS33, both of which were derivatives of RSF1010, had been shown to be transferred by conjugation from *E. coli* into *C. tepidum* [14]. Km and Ap had been used as antibiotics in order to select their *C. tepidum* transconjugants at the concentration of 25–50 and 3 µg/mL, respectively. However, we could not screen out a large number of spontaneous resistant colonies, even at antibiotic concentrations of 100 µg/mL. Any other antibiotic markers were therefore required for the clear-cut selection of the transconjugants under our experimental conditions. We modified pDSK519 to confer the Em- or Sm/Sp-resistance property, whose antibiotics were shown to be effective in the natural transformation experiments [11], and constructed two kinds of pDSK519-derivative plasmids. One is pDSK5191 (Figure 1A), in which the Em-resistance gene, *ermC*, was incorporated with the Ω-T4 phage transcription-translation terminator [23]. The other is pDSK5192, which is almost the same as pDSK5191 except that the Sm/Sp-resistance gene, *aadA*, was incorporated instead of the *ermC* gene (for construction details, see Materials and Methods). The conjugative transfers of these two plasmids were thus reexamined in the presence of Em- and Sm/Sp antibiotics at specific concentrations, respectively.

pDSK5191 and pDSK5192 were introduced into the *C. tepidum* wild-type and ∆*recA* mutant strains by biparental mating with the *E. coli* S17-1 strain [14,21]. Table 3 shows the conjugation frequencies under various mating conditions. When the donor harbored no plasmid, none of colonies that included spontaneous resistant clones appeared on the selective CP plates containing 1.0 µg/mL of Em or 100/50 µg/mL of Sm/Sp. However, when the donor harboring pDSK5191 or pDSK5192 was used, the conjugation resulted in the appearance of transconjugant colonies at the frequency of ~1.0×10^-6^ per total recipient cells. All of the transconjugants were viable and grew actively when they were subcultured on the selective CP plates and/or subinoculated to the liquid CL medium containing the antibiotics. Contamination of the donor *E. coli* S17-1 cells was less than 1.0 cfu/mL, even in the liquid CL culture inoculated just after the first selection, as judged by plating the CL culture to the LB plate. Essentially the same results were obtained from both the *C. tepidum* wild-type and the ∆*recA* mutant strains [13] as recipients. This clearly indicates that pDSK5191 and pDSK5192 can be introduced into the *C. tepidum* cells through conjugation with *E. coli* by screening with appropriate antibiotic selection markers.

**Table 3:** Conjugation frequencies of *C. tepidum* and *C. limnaeum* with *E. coli* S17-1.

**Recipient^a^**	**Plasmid^b^**	**Antibiotics for selection**	**Conjugation frequency^c^**
*C. tepidum* WT2321	-	Em 1.0 µg/mL	< 10^-10^
	pDSK5191	Em 1.0 µg/mL	1.6 ± 0.8 ×10^-5^
	-	Sm/Sp 100/50 µg/mL	< 10^-10^
	pDSK5192	Sm/Sp 100/50 µg/mL	1.2 ± 0.5 ×10^-6^
*C. tepidum ΔrecA*	-	Em 1.0 µg/mL	< 10^-10^
	pDSK5191	Em 1.0 µg/mL	1.7 ± 1.0 ×10^-5^
	-	Sm/Sp 100/50 µg/mL	< 10^-10^
	pDSK5192	Sm/Sp 100/50 µg/mL	6.6 ± 0.2 ×10^-6^
*C. limnaeum* RK-j-1	-	Sm/Sp 100/150 µg/mL	< 10^-8^
	pDSK5192	Sm/Sp 100/150 µg/mL	7.5 ± 3.8 ×10^-5^

^a^ The green sulfur bacterial strains used as the recipients in conjugation experiments. WT2321 and RK-j-1 were used as the wild-type strains of *C. tepidum* and *C. limnaeum*, respectively.

^b^ Plasmids by which green sulfur bacteria were transformed. Hyphens mean that *E. coli* S17-1 without conjugation plasmids was used as a donor.

^c^ Conjugation frequency was calculated as a ratio of colony numbers on the selective plate to that on the non-selective plate. For details, see ‘Materials and Methods’. The detection limit of this method would be 10^-10^ and 10^-8^ for *C. tepidum* and *C. limnaeum*, respectively, because the colony-forming unit (cfu) of CL cultures of the WT2321 and the RK-j-1 strains were approximately 10^10^ and 10^8^ cfu/mL, respectively.

**Figure 2:**
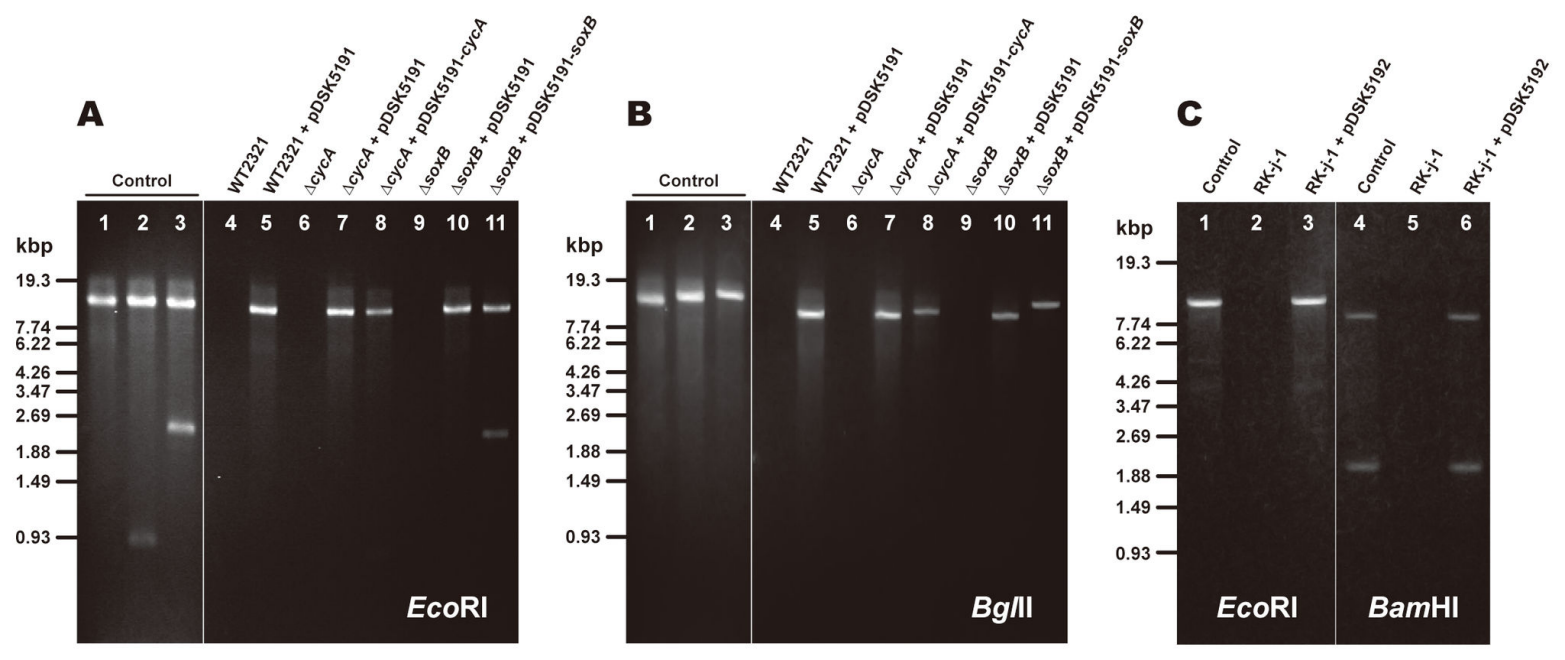
Restriction enzyme mappings of the plasmids in the *C. tepidum* and *C. limnaeum* transconjugants. (A, B) Physical maps of the plasmids from the *C. tepidum* transconjugants. The maps were constructed with restriction enzymes *Eco*RI (A) and *Bgl*II (B). The restriction fragments were separated by agarose gel (1%) electrophoresis. The control plasmids pDSK5191 (lane 1), pDSK5191-*cycA* (lane 2), and pDSK5191-*soxB* (lane 3) were obtained from the donor S17-1 cultures. Lanes 4-11 are the plasmid samples of the *C. tepidum* cultures. The genotype of *C. tepidum* is indicated above each lane. The bars and numbers at the left side of the panel indicate mobility and size of the *Sty*I digests of the λ-phage DNA. (C) Physical maps of the plasmids from *C. limnaeum* transconjugants. The maps were constructed with restriction enzymes *Eco*RI (lanes 1–3) and *Bam*HI (lanes 4–6). Lanes 1 and 4 are pDSK5192 plasmids prepared from the donor S17-1 cultures. Lanes 2-3 and 5-6 are the plasmid samples of the *C. limnaeum* cultures. The genotype of *C. limnaeum* is indicated above each lane. The bars and numbers at the left side of the panel indicate mobility and sizes of the *Sty*I digests of the λ-phage DNA, respectively.

The stable plasmid maintenance in *C. tepidum* transconjugants was confirmed by restriction enzyme mapping of the plasmid (Figure 2). pDSK5191 and its derivatives, pDSK5191-*cycA* and pDSK5191-*soxB*, were introduced into *C. tepidum* by conjugation with *E. coli* S17-1. Plasmids in the transconjugants were extracted from their liquid CL cultures and mapped with the restriction enzymes *Eco*RI and *Bgl*II. No plasmids were extracted from the cultures of the host strains, and 9.6-kbp fragments attributable to the *Eco*RI fragment of pDSK5191 were clearly observed in all the plasmid samples from the transconjugants (Figure 2A, lanes 4–11). Although the small *Eco*RI fragment of *cycA* (0.9 kbp) excised from pDSK5191-*cycA* was not visible well because of the low gel resolution used in this study, the *soxB* fragment (2.4 kbp) from pDSK5191-*soxB* could be detected (Figure 2A, lanes 8 and 11). On the other hand, *Bgl*II mapping clearly showed larger fragments increased by 0.9 and 2.4 kbp that were attributable to the *Bgl*II digests of pDSK5191-*cycA* and pDSK5191-*soxB*, respectively, as indicated in Figure 2B, lanes 8 and 11. The physical mapping of their plasmids remained unchanged after at least five generations of subculturing, and no point mutation was found in the Em-resistance cassettes and the inserted fragments between the *Eco*RI sites as well when their DNA sequences were analyzed. The transconjugants, even after their long-term (over a year) storage in a frozen state, harbored plasmids stably. Therefore, the conjugative plasmid pDSK5191 and its derivatives can be stably maintained in *C. tepidum* cells as long as appropriate antibiotics are applied as selection markers. However, the plasmid pDSK5191-*6xhis-pscAB* could not be introduced into the wild type but into the ∆*recA* mutant as mentioned later.

### Conjugative Plasmid Transfer to *C. limnaeum*

Versatility of the conjugative plasmid pDSK519 derivative was assessed by the conjugation experiments using other green sulfur bacterial species as a recipient strain. The green sulfur bacterium *C. limnaeum* accumulates BChl *e* in its chlorosomes instead of BChl *c* [19]. The biparental mating of *C. limnaeum* and the *E. coli* S17-1 harboring pDSK5192 resulted in the appearance of transconjugant colonies on the selective plates containing 100/150 µg/mL of Sm/Sp at the frequency of ~1.0×10^-4^ per total recipient cells (Table 3). No colony was observed on the selective plates when the *E. coli* S17-1 harboring no plasmid was used as the donor strain. Plasmids in the transconjugants were extracted from their liquid CL cultures and mapped with the restriction enzymes *Eco*RI and *Bam*HI. No plasmids were extracted from the cultures of the host strains (Figure 2C, lanes 2 and 5). In the *Eco*RI mapping, only the 10.4-kbp fragment of pDSK5192 was observed in the plasmid sample from the transconjugant (Figure 2C, lane 3), while the *Bam*HI digestion of pDSK5192 from the transconjugant sample clearly revealed the presence of 8.4- and 2.0-kbp fragments (Figure 2C, lane 6). Subcultures of the transconjugant even after its long-term (over a half year) storage in a frozen state exhibited no significant differences in these results. The plasmid extraction and restriction enzyme mapping thus confirmed stable maintenance of pDSK5192 in *C. limnaeum* RK-j-1.

### Application to Plasmid Complementation

**Figure 3:**
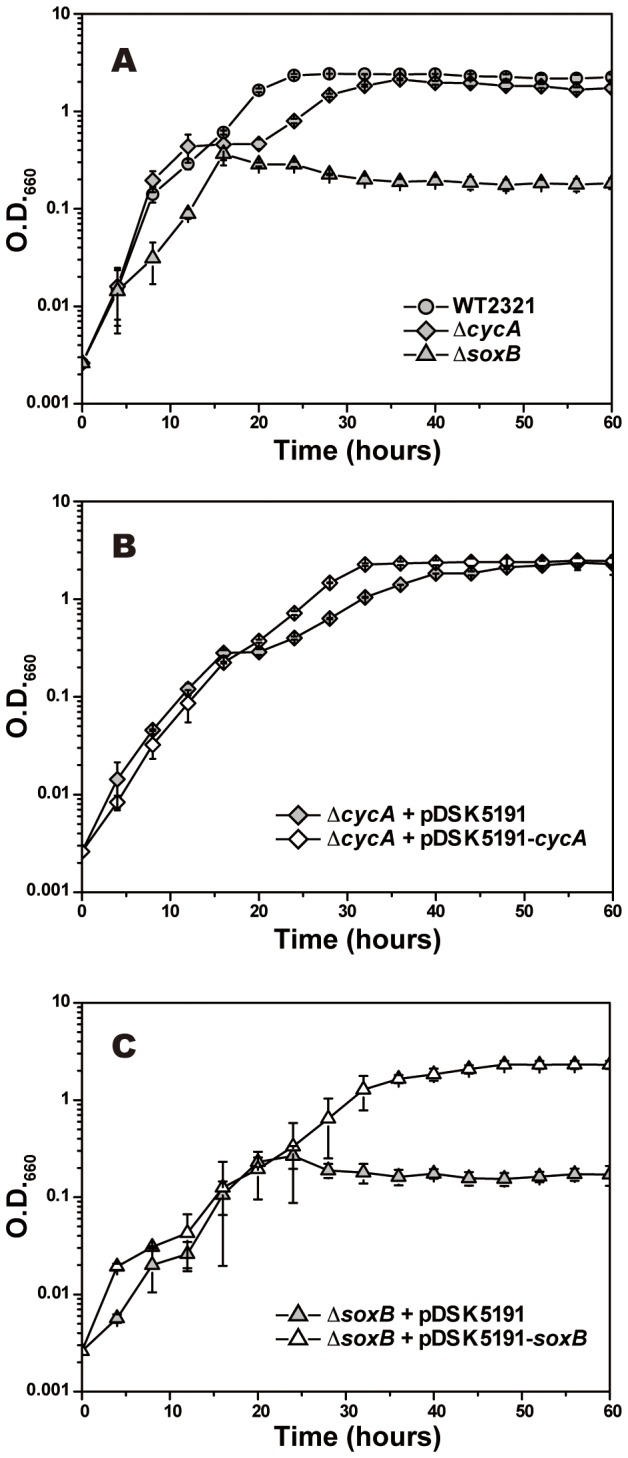
Growth curves of *C. tepidum* mutants after gene complementation experiments. Growth curves of *C. tepidum* mutants used as host strains (A), transconjugant strains of ∆*cycA* mutant (B), and ∆*soxB* mutant (C). Each strain was grown in a liquid CL medium at 40°C (for details, see Materials and Methods), and its optical density (O.D.) was monitored at 660 nm. In the transconjugant cultures, 1 µg/mL of Em was added for the stable maintenance of plasmids. The average values and standard deviations, which were obtained from at least three independent experiments, were plotted.

In order to apply the conjugative plasmid transfer for gene expression experiments in *C. tepidum*, we constructed a pDSK5191-derivative expression plasmid by incorporating a constitutive promoter, P_*pscA*_, and its authentic terminator (Figure 1B) (see Discussion). The availability of this plasmid was examined by gene complementation experiments using the *C. tepidum* knock-out mutant strains ∆*cycA* [28] and ∆*soxB* [5], both of which have characteristic growth phenotypes as shown in Figure 3A when monitored in the liquid CL cultures by measuring O.D._660_: The ∆*cycA* mutant retarded its growth in the mid-to-late-exponential growth phase as compared to the wild-type, and the ∆*soxB* mutant ceased to grow completely before moving into the mid-exponential phase [5]. Their phenotypes have been attributed to the defection of thiosulfate oxidation caused by the disruption of *cycA* and *soxB* genes, respectively [5,29]. Therefore, they would recover active growth behavior if the pDSK5191-derivative plasmids incorporating the relevant genes are maintained stably and work well in the *C. tepidum* cell.

The result of the plasmid complementation of the ∆*cycA* mutant is shown in Figure 3B. pDSK5191 was used as a control vector, and its transconjugant strain was dealt with as a negative control for the growth analyses. This control strain, however, grew somewhat slower than did the original ∆*cycA* strain, presumably because of the presence of Em, but it still exhibited the characteristic retarded growth rate in a mid-to-late-exponential phase. In contrast, the transconjugant strain with the pDSK5191-*cycA* plasmid, which was constructed to express the *cycA* gene (Figure 1B), did not show such growth retardation and exhibited almost the same growth behavior as the wild type during the exponential-growth phase. This indicates that the complementary *cycA* gene in the plasmid could be expressed appropriately and the growth defection of the ∆*cycA* mutant rescued. The same result was also obtained in the complementation experiment of the ∆*soxB* mutant (Figure 3C). The control strain harboring plasmid pDSK5191 exhibited a slightly extended lag phase, but it resulted in almost the same level of growth as compared to that of the ∆*soxB* mutant. The transconjugant strain with the pDSK5191-*soxB* plasmid, on the other hand, grew at an almost constant rate, even after the mid-exponential phase, and attained full growth comparable to the wild-type strain. 

Detailed growth profiles are summarized in Table 4. The wild-type strain harboring the pDSK5191 plasmid grew at a rate approximately 1.5 times slower than that of the original host strain. Presumably, this is because Em provides a somewhat suppressive effect on the growth activity of *C. tepidum*, even if the transconjugant cell has the resistant nature against Em by the plasmid. The strains harboring the pDSK5191 plasmid were thus used as control strains in order to evaluate the growth rates of transconjugants. Strains of the ∆*cycA* and ∆*soxB* mutants complemented by plasmids pDSK5191-*cycA* and -*soxB*, respectively, showed growth rates and final cell yields comparable to those of the wild-type strain with pDSK5191, indicating that the growth defect phenotypes of the mutants were fully rescued by the introduction of their plasmids. This result implies that target genes incorporated into plasmid pDSK5191 are controlled by the constitutive promoter P_*pscA*_ and can be expressed to recover the original phenotypes. Therefore, the plasmid construction as depicted in Figure 1 would be applicable to a range of complementation experiments in *C. tepidum*.

**Table 4:** Growth profiles of the *C. tepidum* transconjugants.

**Host strain**	**Plasmid** ^a^	**Antibiotics** ^b^	**Doubling time (hours)** ^c^	**Cell yield (O.D._660_)** ^c^
WT2321	-	-	2.1 ± 0.0	2.23 ± 0.14
	pDSK5191	Em (1 µg/mL)	3.3 ± 0.2	2.38 ± 0.01
∆*cycA*	-	-	4.5 ± 0.2^d^	1.73 ± 0.15
	pDSK5191	Em (1 µg/mL)	5.0 ± 0.2^e^	2.29 ± 0.61
	pDSK5191-*cycA*	Em (1 µg/mL)	3.6 ± 0.4^f^	2.45 ± 0.16
∆*soxB*	-	-	2.3 ± 0.1	0.18 ± 0.02^d^
	pDSK5191	Em (1 µg/mL)	3.0 ± 0.2	0.17 ± 0.04^e^
	pDSK5191-*soxB*	Em (1 µg/mL)	4.0 ± 0.6	2.31 ± 0.21^f^

^a^ Plasmids introduced by conjugation with *E. coli* S17-1. Hyphens mean that no plasmid was introduced.

^b^ Antibiotics added to the growth media. Hyphens mean that no antibiotic was added.

^c^ Average values and standard deviations of doubling times and cell yields were obtained from at least three independent experiments.

^d^ *P* < 0.01 (*t*-test), by comparison with the value of the WT2321 strain .

^e^ *P* < 0.01 (*t*-test), by comparison with the value of the WT2321 strain with pDSK5191.

^f^ *P* < 0.01 (*t*-test), by comparison with the value of the same host strain with pDSK5191.

### Application to Protein Productions

The availability of the present plasmid construction for protein productions was evaluated by examining the expression level of the His-tagged RC core-polypeptide (PscA). The expression plasmid pDSK5191-*6xhis-pscAB* (Figure 1B), which has the gene cluster of *6xhis-pscA* and *pscB* resulting in the production of the RC complex with His-tagged PscA, was introduced into the *C. tepidum* ∆*recA* mutant. It is noteworthy that no transconjugant colony was obtained when using a host strain with a functional *recA* gene, probably due to defection of the normal chromosomal division by homologous recombination between the *6xhis-pscAB* on the plasmid and the authentic *pscAB* on the chromosome as discussed later [13].

**Figure 4:**
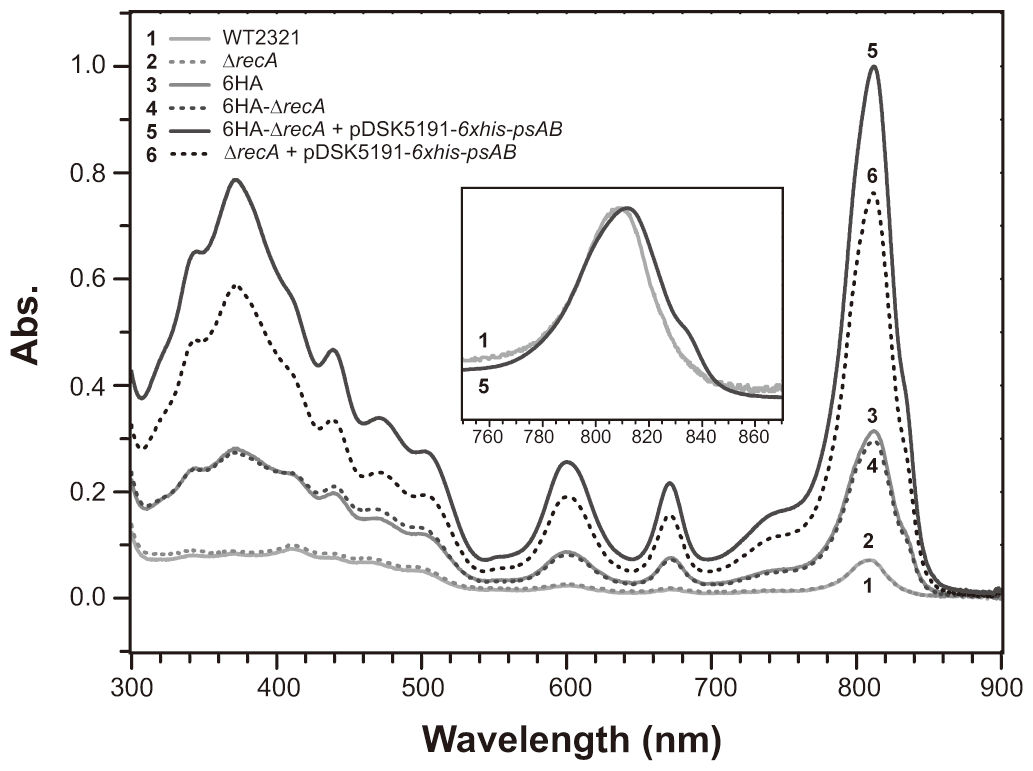
Absorption spectra of BChl *a*-associated proteins from various *C. tepidum* strains recovered by Ni^2+^-affinity purification. Absorption spectra (traces 1-6) of BChl *a*-associated proteins, which were obtained from various *C. tepidum* strains with Ni^2+^-affinity chromatography purification procedure, were measured (see Materials and Methods). Each spectrum was normalized so that recovery rates per wet cell weights can be compared. In the inset, spectra of the eluates from the wild-type WT2321 strain (trace 1) and the 6HA-∆*recA* strain with pDSK5191-*6xhis-pscAB* (trace 5) were normalized at absorption peaks around 810 nm. The absorption band around 810 nm is contributed by antenna BChls a associated with FMO proteins and the RC complex as well, while the absorption shoulder around 840 nm is specific to a special pair of BChls *a*, P840, within the RC complex [3,13].

The expression of His-tagged PscA was confirmed by Ni^2+^-affinity purification of the His-tagged RC complex (Figure 4). The eluates obtained from the wild-type and ∆*recA* mutant strains were pale green and showed their Q_y_-peak wavelengths at 809 nm (Figure 4, traces 1 and 2). As these spectra did not have any absorption shoulder around 840 nm (Figure 4, inset), which serves as a criterion for the presence of the RC complex [3,13], their eluates would contain FMO proteins adsorbed nonspecifically to the Ni^2+^ resin. On the other hand, the specific adsorption of the His-tagged RC complex was observed in the case of the *C. tepidum* 6HA strain (Figure 4, trace 3), whose authentic *pscA* gene on the chromosome was replaced with the *6xhis-pscA* gene (see Materials and Methods). The same result was obtained in the case of the 6HA-∆*recA* strain (Figure 4, trace 4), which was a *recA*-deletion mutant derived from the 6HA strain. When introducing plasmid pDSK5191-*6xhis-pscAB* into the ∆*recA* mutant (Figure 4, trace 6), the specific adsorption of the His-tagged RC complex was also observed, and in significantly larger amounts than that of the 6HA and 6HA-∆*recA* strains. Since the eluate from the ∆*recA* mutant did not contain any His-tagged RC complex, this result clearly indicates that the His-tagged PscA polypeptide was stably expressed from plasmid pDSK5191-*6xhis-pscAB* in the ∆*recA* mutant cells. Moreover, much larger amounts of the His-tagged RC complex were obtained by introducing plasmid pDSK5191-*6xhis-pscAB* into the 6HA-∆*recA* strain (Figure 4, trace 5). There was still a significant increase in the amount of the His-tagged RC complexes recovered from the 6HA-∆*recA* strain with plasmid pDSK5191-*6xhis-pscAB* as compared to the strain from the ∆*recA* mutant with the same plasmid, indicating that the *6xhis-pscA* gene on the chromosome never suppressed the expression of its counterpart on the plasmid.

In order to compare the expression levels of His-tagged PscA in various strains with and without plasmid pDSK5191-*6xhis-pscAB*, we estimated the amounts of total and His-tagged RC complexes that were recovered after solubilization of their membranes and a subsequent procedure with simple and small-scale Ni^2+^-affinity purification, respectively (Table 5). The amounts of the RC complexes were determined from the chemically induced redox difference spectra of P840, which is a special pair of BChls *a* serving as the primary donor in the green sulfur bacterial RC (Table 5, third column) [3,13]. In this case, the amounts of total RC complexes were considered to reflect the relative contents *in vivo* on the assumption that the yields for solubilization of the membranes from various strains were the same. The introduction of pDSK5191-*6xhis-pscAB* to the ∆*recA* mutant caused approximately 35% decrease in the total amount of the RC complex as compared to the host strain. This might imply two things: (1) The total amount of the RC complex is strictly regulated, and the wild-type strain corresponds to its maximum. (2) Coexistence of non- and His-tagged PscAs has a negative effect on the stable assembly of the RC complex in the cell. In fact, the total amount of the RC complexes in both the 6HA and 6HA-∆*recA* strains decreased to approximately 20% of that of the wild-type strain. This would rather imply that His-tagged PscA is less stable than the non-tagged authentic PscA in the *C. tepidum* cell; otherwise some polar effect might arise on the transcription of the *6xhis-pscA* gene by the *aadA* cassette located between the promoter and the Shine-Dalgarno sequence of the *pscA* gene (see Materials and Methods). The introduction of plasmid pDSK5191-*6xhis-pscAB* increased the total amount of the RC complexes to be approximately four times higher as compared to that of the host 6HA-∆*recA* strain. 

**Table 5:** Relative contents of total RC complexes in various *C. tepidum* strains and relative recovery rates of His-tagged complexes.

**Host strain**	**Plasmid^a^**	**Relative contents of total RC complexes^b^**	**Relative recovery rates of the His-tagged RC complex^c^**
WT2321	-	1.00 ± 0.15	N.D.^d^
Δ*recA*	-	1.13 ± 0.22	N.D.^d^
	pDSK5191-*6xhis-pscAB*	0.66 ± 0.12^e,f^	2.91 ± 0.46^f^
6HA	-	0.21 ± 0.04^e^	1.00 ± 0.08
6HA-Δ*recA*	-	0.19 ± 0.05^e^	0.78 ± 0.11
	pDSK5191-*6xhis-pscAB*	0.75 ± 0.09^e,f^	3.93 ± 0.74^f^

^a^ Plasmids introduced by conjugation with *E. coli* S17-1. Hyphens mean that no plasmid was introduced.

^b^ Contents of total RC complexes in various *C. tepidum* strains were represented as ratios to that in the WT2321 strain. Average values and standard deviations were obtained from at least three independent cultures.

^c^ Amounts of His-tagged RC complexes recovered with the Ni^2+^-affinity chromatography purification procedure were represented as ratios to that obtained from the 6HA strain. Average values and standard deviations were obtained from at least three independent cultures.

^d^ ‘N.D.’ means that specific adsorption of the His-tagged RC complex to the Ni^2+^-resin was not detectable.

^e^ *P* < 0.05 (*t*-test), by comparison with the value of the WT2321 strain.

^f^ *P* < 0.01 (*t*-test), by comparison with the value of the 6HA and 6HA-∆*recA* mutant strain.

Additional clear results were provided by comparing the relative recovery rates of the His-tagged RC complexes using the Ni^2+^-affinity purification procedure (Table 5, right column). The introduction of plasmid pDSK5191-*6xhis-pscAB* increased the amount of the His-tagged RC complex recovered from the host 6HA-∆*recA* approximately five times. The recovery rate obtained from the ∆*recA* strain with pDSK5191-*6xhis-pscAB* was also approximately four times higher than that of the 6HA-∆*recA* strain. Since the estimates of the ∆*recA* and 6HA-∆*recA* transconjugant strains with pDSK5191-*6xhis-pscAB* were statistically different (*P* < 0.05, *t*-test), the difference in these estimates would reflect the contribution of the chromosomal expression of His-tagged PscA in the latter strain. 

## Discussion

The first report concerning conjugative plasmid transfer from *E. coli* to *C. tepidum* was published in 1995 [14]. However, we had failed to obtain any transconjugants harboring intrinsic plasmids due to the incomprehensible modifications or rearrangements induced by unknown reasons, even if they had an expected resistant phenotype to antibiotics. As demonstrated in this study, pDSK519-derivative plasmids were reproducibly transferred to *C. tepidum* by mating with *E. coli* S17-1 and were stably maintained in a host cell as well (Table 3 and Figure 2). We conducted the conjugation experiments in almost the same manner as described previously, except for using antibiotics other than Km and Ap [14]. Reexamining the antibiotic selection markers revealed that neither Km nor Ap, used in the original report, was appropriate for screening out spontaneous resistant clones. Instead, Em and Sm/Sp were found to provide complete selection markers for obtaining desirable transconjugants. Strain WT2321 of *C. tepidum* used in our experiments, which was isolated as a plating derivative forming the largest colonies, has been widely used as a model organism for molecular genetic studies of green sulfur bacteria [13]. One possibility might be that the difference in such antibiotic sensitivity arose from some kind of microevolution and/or acclimation of strain WT2321 during laboratory cultivation. In fact, the sensitivity difference we have experienced here was almost identical to that observed in natural transformation experiments [11], suggesting that *C. tepidum* might have acquired Km- and Ap-resistant phenotypes with relative ease under various cultivation conditions. The results mentioned above thus indicate that our methods of selecting the same antibiotics in our conjugation experiments have proved to be as rational as the ones confirmed generally to be effective for natural transformation experiments by different research groups [5,16,28,30]. Otherwise, antibiotic sensitivity should have been checked carefully in order to avoid spontaneous resistant clones.

The experiments with both Em and Sm/Sp selection markers constantly resulted in the formation of transconjugant colonies at almost the same conjugation frequencies (Table 3), although the sensitivity to these antibiotics was significantly different. The concentration required for the complete selection of transconjugants was only 1 µg/mL for Em, while it was at least 100/50 µg/mL for Sm/Sp, respectively. However, a more critical difference between them would be their mechanisms of action that confer antibiotic resistance on host cells. Modifications of Sm and Sp by aminoglycoside 3”-adenylyltransferase, which is the product of *aadA* gene [23], neutralize their antibiotics. Therefore, long-term cultivation could decrease the concentrations of unmodified Sm and Sp in the cultures and might cause the loss of plasmids in transconjugants. In contrast, resistance to Em is based on the dimethylation of an adenine residue in 23S rRNA, which is catalyzed by the product of *ermC* gene, 23S rRNA (adenine-*N* ^*6*^-)-methyltransferase [31], resulting in reduced affinity between Em and ribosomes. Since Em itself is not degraded and works effectively during cultivation, it would therefore be more suitable for strict selection of the *C. tepidum* transconjugants and stable maintenance of the plasmids as well, compared to Sm/Sp.

In the present study, pDSK5191 and pDSK5192 were transferred to the *C. tepidum* wild-type and ∆*recA* mutant strains. Although their plasmid stability was confirmed using only the wild-type strain by restriction enzyme mappings (Figure 2), they would be stably maintained even in the ∆*recA* mutant. In fact, when pDSK5191-*6xhis-pscAB* was transferred into the 6HA-∆*recA* strain as a host cell, the transconjugant could produce three- to four-times higher amounts of His-tagged RC complexes. The His-tagged RC complex thus obtained was indeed an intact one as analyzed with biochemical and spectroscopic methods (a manuscript are now in preparation and will be submitted elsewhere). However, one should pay attention to lengths of homologous regions that plasmids contain. It has been suggested that more than 500-bp homologous flanking regions at both ends of the inserted DNA fragment are indispensable for double-crossover recombination in *C. tepidum* [11]. Therefore, the transconjugant harboring pDSK5191-*6xhis-pscAB*, which contained a 3.5-kbp homologous region to the *pscAB* gene cluster on a chromosome, was never transferred into the *recA* ^+^ strain. On the other hand, pDSK5191-*cycA* and pDSK5191-*soxB*, both of which contained homologous flanking regions of 450-bp P_*pscA*_ and 100-bp terminator, were stably maintained in ∆*cycA* and ∆*soxB* strains, respectively (Figure 2). Although these strains were obtained by insertional inactivation of the relevant genes [5,29], homologous regions left on a chromosome were too small (less than 500 bp) and their recombination with the *cycA* and *soxB* genes on plasmids were never observed. We have not investigated in detail how much the frequency of recombination is dependent on lengths of homologous regions. However, no serious problem seems to be occurred as far as their lengths were less than 500 bp [11].

Both pDSK5191 and pDSK5192 were designed to express target genes by incorporating an approximately 450-bp upstream region of the *pscAB* gene cluster on the *C. tepidum* genome as a putative promoter (P_*pscA*_) region (see Figure 1B). The same region containing P_*pscA*_ was also adopted in the experiment to express the *6xhis*-*pscA* gene that was inserted into a coding region of the *recA* gene [13]. However, the promoter activity of P_*pscA*_ was uncertain in that experiment because the artificially duplicated *6xhis*-*pscA* gene might have been transcribed with an authentic promoter of the *recA* gene regardless of whether the P_*pscA*_ region was functional. In the present plasmid construct, the P_*pscA*_ was located just downstream of the Ω-sequence that terminates transcription from any upstream promoters (Figure 1B). The experimental results of gene complementation (Figure 3), as well as protein expression (Figure 4), clearly indicate that P_*pscA*_ serves as a constitutively active promoter, and that target genes could work without any external signals and/or inducers. Although the product of the *pscA* gene was potentially toxic to *E. coli* in our previous study [13], the P_*pscA*_ region integrated into the expression plasmid as a promoter seems to be not toxic—or less active—in *E. coli* cells. This plasmid construct would facilitate genetic manipulation of target genes even if their products were toxic to *E. coli*. 

There have been two reports so far that natural transformation was used to express chromosome-integrated genes in *C. tepidum*. One is the expression of the heterologous *cruB* gene on the *bchU* locus under control of its promoter [12]. Another is our previous demonstration that a coding region of the *recA* gene served as a neutral site for the insertion of target genes concomitantly with its disruption, resulting in the loss of recombination ability [13]. Nevertheless, the plasmid conjugation method should be advantageous over the conventional natural transformation method. First, plasmid usage is a convenient and quick way to obtain *C. tepidum* strains expressing target genes. The genomic recombination by natural transformation inevitably takes about one month for complete segregation of the mutant allele [11]. In contrast, gene expression by plasmid would take only one week because no segregation process is involved for isolating transconjugants. 

Second, the higher expression level of target genes could be accomplished with plasmids. Growth defects of the ∆*cycA* and ∆*soxB* mutants were fully rescued by expression of their complementary genes with pDSK5191-based plasmid (Table 4). Both products by the *cycA* and *soxB* genes have been recognized as abundant proteins under a sulfide-deficient and thiosulfate-rich condition usually attained in a mid-to-late-exponential-growth phase in the CL culture [32,33], suggesting that gene expressions by pDSK5191 could provide sufficient amounts of proteins to rescue defective mutants. In fact, three- to four-times higher amounts of His-tagged RC complexes were obtained by pDSK5191 expression than those expressed on the chromosome with the same promoter sequence (Table 5). Such an enhancement of gene expression level may simply reflect the increase of a copy number of genes, since the same promoter was utilized to express those genes. A copy number of IncQ-group plasmids is unknown in *C. tepidum* but is supposed to be 9–12 copies, as is the case in *E. coli* and *Pseudomonas aeruginosa* [15]. Thus, a copy number of a chromosome in the *C. tepidum* cell might be estimated to be two to four on average throughout cultivation, although chromosomal ploidy of bacteria generally depends on their growth stages and culture conditions [34]. In addition, higher levels of protein production could be expected by incorporating a more active promoter than P_*pscA*_. Promoters of the *csmA* and *fmoA* genes, whose products were among the most abundant proteins as revealed by the quantitative proteomic analysis of *C. tepidum* [32,35], might be promising candidates for this purpose.

*C. tepidum* is a strictly anaerobic organism, and its growth rate is very fast even at 37–40°C (Table 4), a temperature about 10°C lower than the optimum growth temperature [36]. Therefore, as the third point, the plasmid conjugation system should also be available for the expression of foreign genes, especially those whose products are too oxygen-sensitive, such as nitrogenase and hydrogenase or their recombinant proteins as well, to be stably accumulated in ordinary aerobic bacteria [37,38]. Moreover, since the original plasmid pDSK519 is derived from the broad-host-range plasmid RSF1010 [22], it would be possible to construct a universal plasmid expression system by modifying its promoter so as to be active in other photosynthetic bacteria, that is, purple bacteria as well as cyanobacteria. Conjugative transfer of the plasmid with oriT, which is the replication origin for the conjugative transfer of IncQ-group plasmids, has recently been reported in another green sulfur bacterium, *C. limnaeum* [39], although it was designed as a suicide vector. In the present study, we introduced pDSK5192 into the *C. limnaeum* cell (Table 3) and confirmed its stable maintenance (Figure 2C). Considering the phylogenetically close relationship of the various green sulfur bacterial species [40], the P_*pscA*_ promoter of *C. tepidum* would be active in *C. limnaeum* and other species as well. 

A recent comparative genomics of green sulfur bacteria have revealed an 11-kbp “*sox* island” flanked by 22–23-bp imperfect inverted repeats in a genome of *Chlorobium phaeovibrioides* DSM 265 [41]. This island harbors genes encoding a transposase, an integrase, and a reverse transcriptase in addition to the *soxJXYZAKBW* gene cluster, and is supposed to have been mobilized by a transposase. If this putative mobile element could be activated, it may become a powerful tool for a gene transfer system. In conclusion, the pDSK5191-based plasmid system would thus be the most reliable and easy to use in *C. tepidum* at present.

## Supporting Information

Figure S1 **Construction schemes of conjugative expression plasmids.** Block arrows and rectangles on circles of plasmids represent protein-coding sequences and other notable features, respectively. Radial lines denote recognition sites of selected restriction enzymes and their arbitrary positions are showed as numbers in parentheses. ‘P_*pscA*_’ and ‘ter’ represent promoter and terminator sequences of the *pscAB* gene cluster.
(DOC)

Table S1 **Primers used for DNA constructions and analytical PCRs.**
(DOC)
