# Supplementary figures and images for: Gene Expression System in Green Sulfur Bacteria by Conjugative Plasmid Transfer
Source: PLoS One. 2013 Nov 27;8(11):e82345. doi: 10.1371/journal.pone.0082345 (PMC3842273; doi:10.1371/journal.pone.0082345)

**
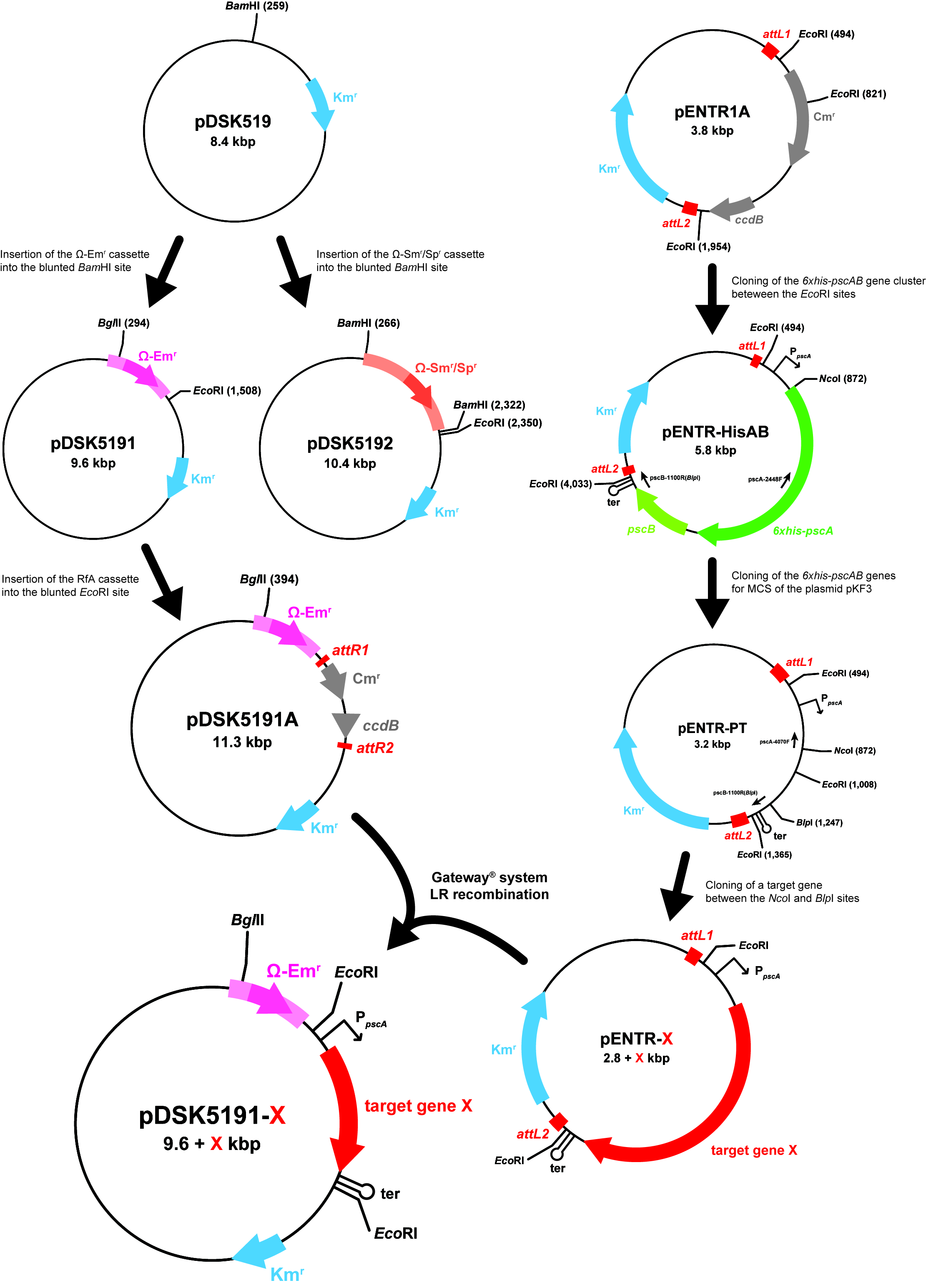
**

**Fgure S1.**

Supplement: Figure S1 — Construction schemes of conjugative expression plasmids. Block arrows and rectangles on circles of plasmids represent protein-coding sequences and other notable features, respectively. Radial lines denote recognition sites of selected restriction enzymes and their arbitrary positions are showed as numbers in parentheses. ‘PpscA’ and ‘ter’ represent promoter and terminator sequences of the pscAB gene cluster. (DOC) [file pone.0082345.s001.doc]
