# Supplementary material for: Gene Expression System in Green Sulfur Bacteria by Conjugative Plasmid Transfer
Source: PLoS One. 2013 Nov 27;8(11):e82345. doi: 10.1371/journal.pone.0082345 (PMC3842273; doi:10.1371/journal.pone.0082345)
Supplement: Table S1 — Primers used for DNA constructions and analytical PCRs. (DOC) [file pone.0082345.s002.doc]

Table S1. Primers used for DNA constructions and analytical PCRs

| Primer name ^a^ | Sequence ^b^ | Reference ^c^ |
| --- | --- | --- |
| dapF-5291R | cacatgcatggttgacggag | This study |
| dapF-5115R (*Sma*I) | aaaCCCGGGcttttgacctttaccctgaca | This study |
| pscA-4435R | ggtgaaatcgatgtgcatgtc | [13] |
| pscA-4095F | tattttcaggttgaagaaaccg | [13] |
| pscA-4094R | cgttcaaacaaaggagaacatac | This study |
| pscA-4072F (*Nco*I) | TCAGCCATGGTATGTTCTCCTTTGTTTGAACG | [13] |
| pscA-4070F | tgtatgttctcctttgtttgaa | This study |
| pscA-3561F | cgtcagattgtgcgtccagt | This study |
| pscA-2448F | GCTTGTCGAGTTCCTTGTAG | This study |
| pscB-1100R (*Blp*I) | CCTGCTgAGCcagatagtcaggaatcg | This study |
| pscB-996F | ttaacagaactggacttgaatc | This study |
| HP45-blaF | CAAGGATCTTACCGCTGTTG | [13] |
| HP45-ropR | GCTTACAGACAAGCTGTGAC | [13] |
| cycA-2684F | atgtcccgtttcgtttcagc | This study |
| cycA-3039R (*Blp*I) | aaaGCTcAGCcccgttttccgctggtatg | This study |
| soxB-4306F | atgtttcgggacgagcctttc | This study |
| soxB-6170R (*Blp*I) | aaaGCTcAGCtgacgatactcgcctcaaga | This study |
| aadA-F2 (*Sma*I) | aaaCCCGGgaacgcagcggtggtaac | This study |
| aadA-R2 (*Sma*I, *Blp*I, *Hin*dIII) | aaaaagcttgctCagcccggGACATTATTTGCCGACTACCTT | This study |
| T7 Promoter primer | taatacgactcactataggg | TaKaRa Bio. |

^a^ Restriction enzyme sites attached to primers for cloning are shown in parentheses.

^b^ Recognition sequences of the restriction enzymes are underlined.

^c^ The number of reference corresponds to that in the bibliography of main text.
